# Minimum inhibitory concentrations of commercial essential oils against common chicken pathogenic bacteria and their relationship with antibiotic resistance

**DOI:** 10.1111/jam.15302

**Published:** 2021-09-28

**Authors:** Nguyen Thi Bich Van, On Thuong Vi, Nguyen Thi Phuong Yen, Nguyen Thi Nhung, Nguyen Van Cuong, Bach Tuan Kiet, Nguyen Van Hoang, Vo Be Hien, Guy Thwaites, James Campell, Marc Choisy, Juan Carrique‐Mas

**Affiliations:** ^1^ Oxford University Clinical Research Unit Hospital for Tropical Diseases Ho Chi Minh City Vietnam; ^2^ International University Vietnam National University Hanoi Vietnam; ^3^ Sub‐Department of Animal Health Dong Thap Province Cao Lanh Vietnam; ^4^ Nuffield Department of Medicine Oxford University Oxford UK

## Abstract

**Aims:**

We investigated the antibacterial effect of seven essential oils (EOs) and one EO‐containing liquid phytogenic solution marketed for poultry and pigs (‘Product A’) on chicken pathogens, as well as the relationship between minimum inhibitory concentration (MIC) in EOs and antibiotics commonly administered to chicken flocks in the Mekong Delta (Vietnam).

**Methods and Results:**

Micellar extracts from oregano (*Origanum vulgare*), cajeput (*Melaleuca leucadendra*), garlic (*Allium sativum*), black pepper (*Piper nigrum*), peppermint (*Mentha* × *piperita* L.), tea tree (*Melaleuca alternifolia*), cinnamon (*Cinnamomum zeylanicum*) EOs and Product A were investigated for their MIC against *Avibacterium endocarditidis* (*N* = 10), *Pasteurella multocida* (*N* = 7), *Ornitobacterium rhinotracheale* (ORT) (*N* = 10), *Escherichia coli* (*N* = 10) and *Gallibacterium anatis* (*N* = 10). Cinnamon EO had the lowest median MIC across strains (median 0.5 mg/ml [IQR, interquartile range 0.3–2.0 mg/ml]), followed by Product A (3.8 mg/ml [1.9–3.8 mg/ml]), oregano EO (30.4 mg/ml [7.6–60.8 mg/ml]) and garlic 63.1 mg/ml [3.9 to >505.0 mg/ml]. Peppermint, tea tree, cajeput and pepper EOs had all MIC ≥219 mg/ml. In addition, we determined the MIC of the 12 most commonly used antibiotics in chicken flocks in the area. After accounting for pathogen species, we found an independent, statistically significant (*p* < 0.05) positive correlation between MIC of 10 of 28 (35.7%) pairs of EOs. For 67/96 (69.8%) combinations of EOs and antibiotics, the MICs were correlated. Of all antibiotics, doxycycline was positively associated with the highest number of EOs (peppermint, tea tree, black pepper and cajeput, all *p* < 0.05). For cinnamon, the MICs were negatively correlated with the MICs of 11/12 antimicrobial tested (all except colistin).

**Conclusions:**

Increases in MIC of antibiotics generally correlates with increased tolerance to EOs. For cinnamon EO, however, the opposite was observed.

**Significance and Impact of the Study:**

Our results suggest increased antibacterial effects of EOs on multi‐drug resistant pathogens; cinnamon EO was particularly effective against bacterial poultry pathogens.

## INTRODUCTION

Essential oils (EOs) are volatile lipophilic substances obtained from plants by cold extraction, steaming or alcohol distillation. Many EOs are used to manufacture products including food flavouring additives, preservatives, cosmetics, detergents and insect repellents. EOs are chemically complex substances and their composition may greatly vary depending on the geographical location and growing conditions of the source plant, as well as the extraction method (Rhind, 2012). Many EOs have the capacity to eliminate/inhibit bacterial, fungal and viral pathogens (Ebani & Mancianti, 2020; Ebani et al., 2018; Swamy et al., 2016), as well as displaying anti‐oxidative and anti‐inflammatory properties. Because of this, EOs have traditionally been used to treat a wide range of human diseases (i.e. aromatherapy). However the use of EOs may also result in adverse health effects (Ramsey et al., 2020).

Antibiotics are extensively used in animal production, both to prevent and treat disease; in many countries they are also added to commercial animal feeds as antimicrobial growth promoters (AGPs) (Pagel & Gautier, 2012). The worldwide emergence of antimicrobial resistance (AMR) and the increased awareness of the role of antimicrobial use (AMU) in animal production (O'Neill, 2015) has led to a renewed interest in the potential of EOs as replacement or adjunct to antibiotics in animal production without compromising human health.

Several studies have recently shown the potential of EOs to improve growth performance in poultry and pigs (Franz et al., 2009; Omonijo et al., 2017; Windisch et al., 2008; Zhai et al., 2018). In addition, the use EOs has been proposed in food production to control foodborne infections such as nontyphoidal *Salmonella* (Bajpai et al., 2012; Dewi et al., 2021; Ebani et al., 2019), *Campylobacter* spp. (Micciche et al., 2019) or *Listeria monocytogenes* (Yousefi et al., 2020). Although there are limited data on the efficacy of EOs against diseases of pigs and cattle (Amat et al., 2019; LeBel et al., 2019), there are virtually no data on the effect of EOs on poultry pathogens, or the relationship between AMR and susceptibility against EOs. One recent study investigated the effect of 16 EOs on one strain of avian pathogenic *Escherichia coli* (APEC) (Ebani et al., 2018). In terms of production, chicken is the most commonly consumed type of meat worldwide (OECD, 2020), and the chicken species is globally the target of the greatest levels of AMU (Cuong et al., 2018). Several bacterial pathogens have been identified in diseased chicken flocks in the Mekong Delta of Vietnam, including *Avibacterium paragallinarum*, *Avibacterium endocarditidis*, *Gallibacterium anatis*, *Mycoplasma gallisepticum* (MG), septicaemic *E. coli* and *Ornithobacterium rhinotracheale* (Van et al., 2020; Yen et al., 2020). Some of these pathogens have been investigated for their susceptibility against the nine most used antibiotics in the area in order to provide treatment guidelines (Yen et al., 2020).

It has been shown that AMR in bacteria is often associated with reduced fitness (i.e. fitness costs) (Bengtsson‐Palme et al., 2018). Therefore, among bacteria resistant to antibiotics we would expect them to display reduced tolerance to EOs (i.e. reflected in a reduced MIC). On the other hand, if the mechanisms of resistance for EOs and antibiotics were related, we would expect a positive association between the MICs of these two types of substances. The aim of this study was to determine the minimal inhibitory concentration (MIC) (*in vitro* effect) of eight commonly available EOs on common pathogenic bacteria isolated from chicken flocks Vietnam, and to investigate the relationship between MICs against antibacterials and EOs in different bacterial species. Results from this study should help identifying which EO/s that may have the potential to replace antibiotics to control infections in poultry production.

## MATERIAL AND METHODS

### Essential oils

Seven EOs were investigated, including those extracted from oregano (*Origanum vulgare*), cajeput (*Melaleuca leucadendra*), garlic (*Allium sativum*), black pepper (*Piper nigrum*), peppermint (*Mentha* × *piperita* L.), tea tree (*Melaleuca alternifolia*) and cinnamon (*Cinnamomum zeylanicum*) (Heber, Ho Chi Minh City, Vietnam). In addition, a commercial liquid phytogenic solution that contains EOs from oregano and cinnamon in its composition and is marketed for poultry/livestock (Product A) was tested. The composition of this product also includes water, pectin, citric acid and sodium chloride. The EOs contents were further investigated for their composition by gas chromatography mass spectrometry (GC/MS) (Quality Assurance and Testing Centre 3). The properties and chemical compositions of the EO formulations investigated are shown in Table S1.

### Bacterial strains

A total of 47 isolates belonging to five different bacterial species were investigated. These included *A. endocarditidis* (*n* = 10), *Pasteurella multocida* (*n* = 7), *Ornitobacterium rhinotracheale* (ORT) (*n* = 10), septicaemic *E. coli* (*n* = 10) and *G. anatis* (*n* = 10). All isolates were recovered from diseased chickens raised in flocks in Mekong Delta of Vietnam. *A*. *endocarditidis*, ORT and *G*. *anatis* strains were recovered from the upper respiratory tract. *Escherichia coli* isolates were recovered from the liver/spleen of septicaemic birds. ORT, *P*. *multocida* and *G*. *anatis* isolates were recovered from blood agar (Oxoid) incubated at 37℃ + 5% CO_2_ for 24 h. *Avibacterium endocarditidis* isolates were recovered using chocolate agar (Oxoid) at 37℃ + 5% CO_2_ for 24 h. Invasive *E*. *coli* and *E*. *coli* ATCC strains were recovered from nutrient agar incubated at 37℃ for 24 h. The species identity of all bacterial strains was confirmed by Matrix‐Assisted Laser Desorption Ionization Time‐Of‐Flight Mass Spectrometry (MALDI‐TOF MS) (Bruker). The ATCC 25922 *E*. *coli* strain was used as control. All bacterial strains were maintained in tryptic soy broth (TSB) medium with glycerin, at −60℃.

### Determination of MIC of EOs

Since EOs are hydrophobic, we processed them to obtain homogenous micelles miscible with water‐based bacterial suspensions (Man et al., 2017, 2019). Suspensions of 2 ml of each EO and sterile water (1:1) were prepared using Eppendorf micro‐centrifuge tubes. Micelles were obtained by sonication at 43 kHz for 20 min at room temperature (~25℃) using a sonicated water bath (DG‐1, MRC Ltd). The bottom homogenous opalescent phase was recovered using fine sterile pipette tips and was used as stock micelle solution.

The MIC of EOs was determined by broth microdilution according to Clinical and Laboratory Standards Institute (CLSI) guidelines (document M07) (CLSI, 2018) using 96‐well plates (Corning). Bacterial inocula were prepared by creating bacterial suspensions in saline solution (0.85% NaCl) adjusted to a turbidity equivalent to 0.5 McFarland (2 × 10^8^ colony forming units/µl). The suspensions were adjusted by diluting in 1:100 sterile cation‐adjusted Mueller Hinton‐II broth (MHB2, Sigma‐Aldrich); for ORT, *P*. *multocida* and *G*. *anatis* 5% lysed horse blood (E&O Laboratories) was added. Fifty microlitres of diluted bacterial suspension and 50 *µ*l of EO dilution were added to each of a 96‐well plate, and twofold serial dilutions were performed. The dilutions tested ranged from ~0.25 to ~500 mg/ml. Plates were incubated for 24 h at 37℃. CO_2_ was added to ORT, *P*. *multocida*, *G*. *anatis* and *A*. *endocarditidis* cultures. In order to correctly interpret the readings from wells containing horse blood, we transferred 10 *µ*l of each well into a new plate containing fresh MH +5% lysed horse blood, incubated for a further day, and then re‐read the results. If these were still unclear, we repeated this step. The MIC value was defined as the lowest concentration at which bacteria showed no growth and was interpreted as v/v percentage of stock solution. All tests were performed in triplicate.

### Determination of MICs of antibiotics

The minimum inhibitory concentration (MIC) of 12 of the most commonly used antibiotics in chicken flocks in the area were investigated by broth micro‐dilution following Clinical Laboratory Standards Institute (CLSI) procedures outlined in VET01S (CLSI, 2015) and M100 (CLSI, 2019). The antibiotic panel included colistin (COL), oxytetracycline (OXY), tylosin (TYL), doxycycline (DOX), gentamicin (GEN), amoxicillin (AMX), enrofloxacin (ENR), neomycin (NEO), streptomycin (STR), florfenicol (FFN), thiamphenicol (THA) and co‐trimoxazole (SXT). The bacterial inocula were prepared as described above. The dilutions tested ranged from 0.03 to 256 µg/ml.

### Data analyses

In order to investigate the association between MICs between different EOs, as well as between EOs and antibiotics, whilst correcting for the potential confounding effects of the pathogens, we built generalised linear models with normal residuals. The MIC value of each EO was specified as outcome (log2 transformed), and ‘pathogen species’ and MIC (log2 transformed) of each of the other EOs and antibiotics as covariates. We computed a correlation coefficient between MICs (corrected for the ‘pathogen species’ effect) as the ratio of the full model's residual deviance to the residual deviance of a model with ‘pathogen species’ only as a covariate. The significance of the correlation was computed by a ratio test on the likelihood of these two models. All analyses were carried out using R software v4.0.3.

## RESULTS

### MICs of EOs and antibiotics

**FIGURE 1:**
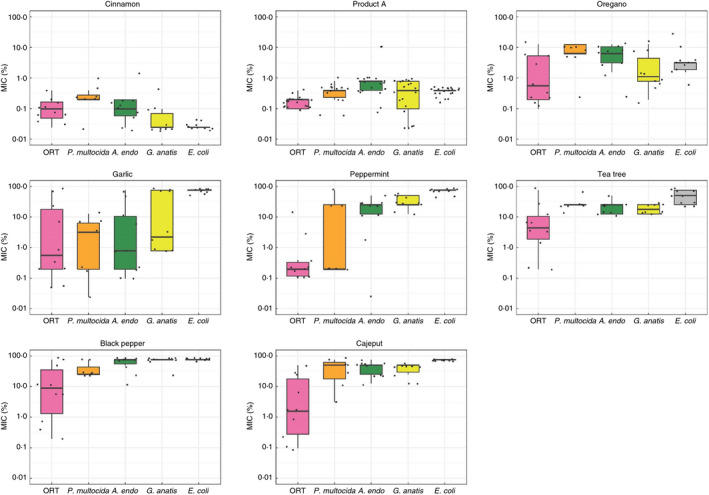
MIC of different EOs against tested bacterial strains

The MIC results of EOs are shown in Table 1 and summarized in Figure 1. Of all EOs investigated, cinnamon EO had the lowest median MIC across strains (median 0.5 mg/ml [interquartile range (IQR) 0.3–2.0 mg/ml]), followed by Product A (3.8 mg/ml [1.9–3.8 mg/ml]), oregano (30.4 mg/ml [7.6–60.8 mg/ml]), garlic (63.1 mg/ml [3.9–750.0 mg/ml]), tea tree (219.8 mg/ml [109.9–219.8 mg/ml]), peppermint (223.0 mg/ml [1.7–446.0 mg/ml]), cajeput (455.0 mg/ml [113.8 to >455.0 mg/ml]) and black pepper (>431.5 mg/ml [215.8–431.5 mg/ml]). Of the pathogens investigated the lowest MIC (i.e. greatest susceptibility) corresponded to ORT (3.5 mg/ml [1.7–54.9mg/ml]), and the highest to *E*. *coli* (627.5 mg/ml [4.8–431.5 mg/ml]). Cajeput, black pepper, peppermint and garlic EOs had no antibacterial activity on *E*. *coli* strains, even at high concentration. MIC results of antibiotics are shown in Table 2. Three *E*. *coli* isolates were not further recovered due to a problem during storage. The full data set is available in Table S3.

**TABLE 1:** Range of MIC (defined as the lowest concentration at which bacteria suspensions showed no growth, incubated at 37℃ for 24 h) values of eight EOs against 47 bacterial strains belonging to five species

		MIC (mg/ml)													
		MIC_50_	0.3	0.5	1.0	2.0	4.1	8.2	16.4	32.8	65.5	131.0	262.0	524.0	>524.0
Cinnamon	AE	1.0	2	1	3	3			1						
	PM	2.0	1			4	1	1							
	ORT	1.0	1	3	3	2	1								
	EC	0.3	8	2											
	GA	0.3	7		2		1								
		MIC_50_	0.2	0.5	1.0	1.9	3.8	7.7	15.3	30.6	61.3	122.5	245.0	490.0	>490.0
Product A	AE	7.7			1		3	5				1			
	PM	3.8		1		1	4	1							
	ORT	1.9			4	5	1								
	EC	3.8				2	8								
	GA	3.8	2		1	1	2	4							
		MIC_50_	0.2	0.5	0.9	1.9	3.8	7.6	15.2	30.4	60.8	121.5	243.0	486.0	>486.0
Oregano	AE	60.8				1			1	2	3	3			
	PM	60.8				1					3	3			
	ORT	5.7			1	3	1	1		1	2	1			
	EC	30.4						1	2	5		1	1		
	GA	11.4				1	1	3	2		2	1			
		MIC_50_	0.2	0.5	1.0	2.0	3.9	7.9	15.8	31.6	63.1	126.3	252.5	505.0	>505.0
Garlic	AE	7.9			2	2		2			1	1		1	1
	PM	31.6	1			2				1	2	1			
	ORT	5.9		2		2	1	1			1		1		2
	EC	>505												2	8
	GA	23.7						4	1	1					4
		MIC_50_	0.2	0.4	0.9	1.7	3.5	7.0	13.9	27.9	55.8	111.5	223.0	446.0	>446.0
Peppermint	AE	223	1						1			2	5	1	
	PM	1.7				4							2		1
	ORT	1.7			3	4	1			1		1			
	EC	>446.0													10
	GA	223										2	4	4	
		MIC_50_	0.2	0.4	0.9	1.7	3.4	6.9	13.7	27.5	54.9	109.9	219.8	439.5	>439.5
Tea tree	AE	219.8										4	5	1	
	PM	219.8										1	5		1
	ORT	41.2				2			1	2	2	1	1		1
	EC	439.5											4	2	4
	GA	164.9										5	5		
		MIC_50_	0.2	0.4	0.8	1.7	3.4	6.7	13.5	27.0	53.9	107.9	215.8	431.5	>431.5
Black pepper	AE	>431.5										1	1	1	7
	PM	215.8											5		2
	ORT	80.9				1	1	1			2	2		1	2
	EC	>431.5													10
	GA	>431.5											1		9
		MIC_50_	0.2	0.4	0.9	1.8	3.6	7.1	14.2	28.4	56.9	113.8	227.5	455.0	>455.0
Cajeput	AE	455.0										1	3	5	1
	PM	455.0								1		1	1	2	2
	ORT	14.2			2	1		1	2		1		2	1	
	EC	>455.0													10
	GA	455.0										2	1	7	

Key: AE = *A. endocarditidis*, PM = *P. multocida*, EC = Invasive *Escherichia coli*, GA = *Gallibacterium anatis*. MIC_50_ = Minimum concentration of EOs that inhibits 50% of strains.

**TABLE 2:** Range of MIC (defined as the lowest concentration at which bacteria suspensions showed no growth, incubated at 37℃ for 24 h) values of 12 antibiotics against 44 strains belonging to five bacterial species

		MIC (µg/ml)														
		MIC 50	0.03	0.06	0.125	0.25	0.5	1	2	4	8	16	32	64	128	256
Florfenicol	AE	0.5					7					3				
	PM	0.5					4				3					
	ORT	0.5					9	1								
	EC	132								1	2	#				4
	GA	0.5					8						1	1		
Colistin	AE	1					2	4	3		1					
	PM	2						1	6							
	ORT	64											2	8		
	EC	1						6	1	#						
	GA	1					1	9								
Co‐trimoxazole	AE	2						4	2		2	2				
	PM	0.125	1	1	3	1	1									
	ORT	5					1	2	2		1	4				
	EC	16						1		#		6				
	GA	4.5	2			1		2			1	4				
Gentamicin	AE	2					1	2	3	2				2		
	PM	4							1	3						3
	ORT	24								2	1	2	5			
	EC	33							3			#		1		3
	GA	0.5					8	2								
Neomycin	AE	6							4	1	1	2	2			
	PM	4								4						3
	ORT	24					1			3		1	5			
	EC	6							2	1	1	#			1	2
	GA	1						6	1		1	2				
Doxycycline	AE	3							5	3		1		1		
	PM	0.5					4					3				
	ORT	3					1	2	2	1	3	1				
	EC	8								1	3	#	3			
	GA	6								5	4	1				
Amoxicillin	AE	4					1	1	2	2	2	1	1			
	PM	1						4				1	2			
	ORT	6					1		1	3	3		2			
	EC	256											#			7
	GA	8					1			1	5	1		1		1
Enrofloxacin	AE	10			2			2		1		2	2	1		
	PM	0.125			7											
	ORT	12			1	2				1	1	3	2			
	EC	24							#			3	2	2		
	GA	12				1	1	1			2	2	1	2		
Streptomycin	AE	12								2	3	1				4
	PM	16									2	2				3
	ORT	16								2	1	5	2			
	EC	256												#1		6
	GA	34							2	3				1	3	1
Tylosin	AE	64										4		2	2	2
	PM	32										2	2			3
	ORT	1					4	5			1					
	EC^a^															
	GA	48										1	4	2	2	1
Oxytetracycline	AE	64										1	3	5		1
	PM	0.5					4								3	
	ORT	2					1	3	2	1	2	1				
	EC	256										#			1	6
	GA	192												2	3	5
Thiamphenicol	AE	256						2								8
	PM	0.75					3	1								3
	ORT	2					1	2	4						3	
	EC	192											1	1	1	4
	GA	64.5					2	3							3	2

# = Breakpoint for phenotypic resistance; MIC_50_ = minimum concentration of EOs that inhibits 50% of strains.

^a^ Not tested.

### Correlations between MICs of EOs

**FIGURE 2:**
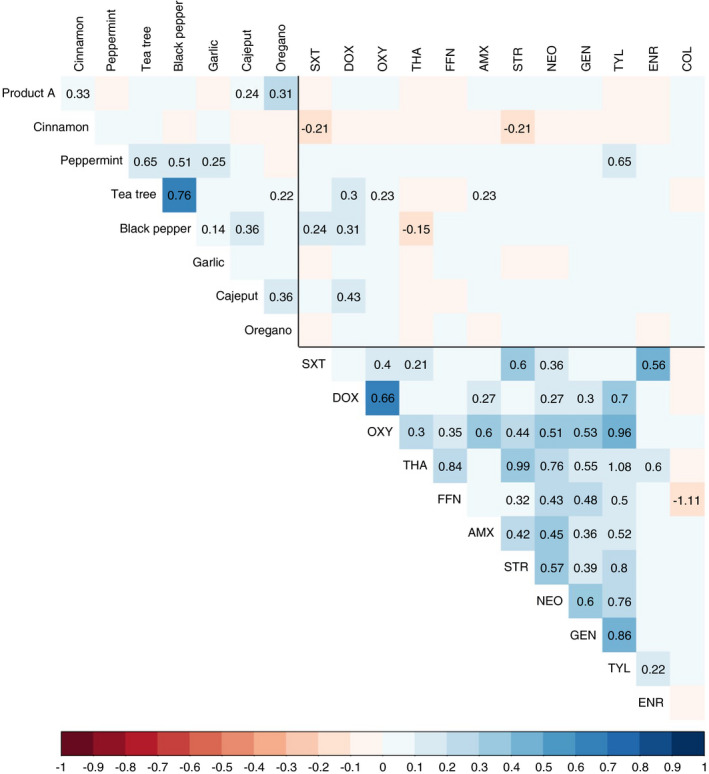
Association between the MICs of antimicrobials and EOs. The intensity of the color indicate the correlation coefficient corrected for the pathogen effect. For significant (*p* < 0.05) correlations, the value of the regression coefficient is also shown. Key: COL = colistin; ENR = enrofloxacin; TYL = tylosin; GEN = gentamicin; NEO = neomycin; STR = streptomycin; AMX = amoxicillin; FFN = florfenicol; THA = thiamphenicol; OXY = oxytetracycline; DOX = doxycycline; SXT = co‐trimoxazole; ORE = oregano; CAJ = cajeput; GAR = garlic; BLA = black pepper; PEP = peppermint; TEA = tea tree; CIN = cinnamon; PRO = Product A

We examined the potential correlations between the MIC of all (28) pair‐wise combinations of the eight EOs investigated. After accounting for pathogen species, we found an independent, statistically significant (*p* < 0.05) positive association for 10/28 (35.7%) combinations (Figure 2). The greatest correlation corresponded to the pairs tea tree and black pepper (0.76), peppermint and tea tree (0.65), and peppermint and black pepper (0.51). The greatest overall variability of the data was due to the EOs (ICC = 0.47), and to a lesser extent, the bacterial species identity (intra‐class correlation coefficient [ICC] = 0.15).

### Correlations between EO and antibiotic MICs

For 67/96 (69.8%) combinations the MICs of EOs were positively correlated with MICs of antibiotics. For the remaining 29 (30.2%) combinations, there were negative correlations. However, only in 10/96 (10.4%) of cases were these correlations statistically significant. For three of those (30%) negative associations were observed: SXT‐cinnamon (−0.21), streptomycin‐cinnamon (−0.21) and thiamphenicol‐black pepper (−0.15). Interestingly, the MICs of cinnamon were negatively correlated with the MICs of 11/12 antibiotics tested (all except colistin). Of all antibiotics, doxycycline was positively associated with the highest number of EOs (peppermint, tea tree, black pepper and cajeput, all *p* < 0.05).

## DISCUSSION

We observed considerable variation in the *in vitro* inhibitory effects of the different EOs investigated. To a lower extent, the observed differences also depended on the pathogen investigated. Cinnamon EO and Product A displayed the highest inhibitory activity against all bacterial species investigated; in contrast, EOs from tea tree, black pepper and cajeput displayed low inhibitory activity.

The main active component of cinnamon EO is cinnamaldehyde. A previous study reported antibacterial activity of EOs from *Cinnamomum burmannii*, with MICs ranging from 0.1 to 8.0 mg/ml for species including *Acinetobacter*, *Klebsiella pneumoniae*, *Proteus vulgaris*, *Enterococcus faecalis*, *Staphylococcus aureus* and *Staphylococcus epidermidis* (Aumeeruddy‐Elalfi et al., 2015). Also, previous studies have documented inhibitory activity of cinnamon EO on biofilm formation of *S*. *aureus* (Nuryastuti et al., 2009), as well as on bacteria causing meat spoilage (Oussalah et al., 2006). Notably, the inhibitory effect of cinnamon EO on invasive *E*. *coli* strain was superior to that of any other EO investigated. Furthermore, cinnamon EO has shown positive effects on broiler growth (Abd El‐Hack et al., 2020).

A study investigating the activity of nine EOs on six major pig pathogens (including *P*. *multocida*) highlighted a relatively lower MIC values for cinnamon (0.0193–0.078%, v/v) compared with peppermint EO (0.078–0.625%) (LeBel et al., 2019). However, in that study only 1–4 isolates of each bacterial species were included.

The MIC values obtained in our study for cinammon EOs against *E*. *coli* strains (median 0.3 mg/ml) were lower than that in other studies (0.6–1.25 mg/ml) (Park et al., 2017; Zhang et al., 2016), but higher than results on a control (ATTC) strain (0.005 mg/ml) (El Atki et al., 2019).

Interestingly, we found more positive than negative correlations between MICs of EOs and antibiotics, suggesting that reduced susceptibility to EOs may be linked to AMR in some cases. In fewer occasions, we found that increased MIC against antibiotics lead to increased susceptibility to EOs (presumably as a result of fitness costs conferred by phenotypic AMR). Notably, increased resistance to several antibiotics was reflected in greater susceptibility to cinnamon EO. Although highly speculative, this suggests that the acquisition of resistance may reduce the bacteria's ability to counter the activity of this EO.

The relatively few isolates investigated limit the interpretability of our results for single bacterial species. However, the isolation of animal pathogens in many low‐ and middle‐income countries (LMICs) is challenging because of limited diagnostic capacity (Gandra et al., 2020). In Vietnam, there are currently very few veterinary laboratories capable of performing diagnostic bacteriology.

EOs are generally less toxic, and therefore would theoretically be optimal alternatives to conventional antibiotics. However, for most EOs the MIC values are higher than for conventional antibiotics, requiring increased strength in feed/water formulations. This poses challenges in terms of palatability and costs. There are also considerable challenges regarding product standardization, since EOs are complex substances, and their composition may greatly vary according to a number of factors.

Recent studies have demonstrated that bacteria exposed to sublethal doses of EOs may result in increased tolerance (Melo et al., 2015). However, this may depend on specific EO‐bacterial combinations (Becerril et al., 2012). In our study, we observed great differences in the MICs of number of EOs against specific bacterial species, notably garlic. More research is needed to determine the development of tolerance of poultry pathogens against EOs. In all cases, the use of EOs in poultry formulations should avoid the inclusion of EOs in sublethal strength. Furthermore, we recommend monitoring the effectiveness of EOs over time.

Many LMICs have begun to draft legislations and policies aiming at restricting the use of antibiotics for prophylaxis and growth promotion. For example, Vietnam introduced in 2018 the Animal Husbandry Law (32/2018/QH14), which included a full ban on AGPs in commercial feeds. A further decree (13/2020/ND‐CP) (Anon., 2020) established a timeframe for banning all prophylactic use of antibiotics, with full bans expected by the end of 2025. Much of the AMU in pig and poultry production in the country is for prophylactic purposes. The upcoming bans make it more pressing to find effective alternatives to antimicrobials in livestock production. Our results suggest that EOs, especially those that contain cinnamaldehyde and carvacrol may efficiently be used to treat bacterial poultry diseases. Further studies are required to establish its optimal concentrations and potential toxicity when included in poultry rations.

## CONFLICT OF INTEREST

No conflict of interest declared.

## Supporting information

Table S1

Table S2

Table S3
