# Supplementary material for: Minimum inhibitory concentrations of commercial essential oils against common chicken pathogenic bacteria and their relationship with antibiotic resistance
Source: J Appl Microbiol. 2021 Sep 28;132(2):1025–35. doi: 10.1111/jam.15302 (PMC9293407; doi:10.1111/jam.15302)
Supplement: Supplementary file 1 — Table S1 [file JAM-132-1025-s003.docx]

**Table S1.** Properties and chemical compositions of essential oils.

| Essential oils | Plant component | Composition |
| --- | --- | --- |
| Oregano  *(Origanum vulgare)* | Herb | Carvacrol (88.6%), Thymol (7.4%), Cymol (2.7%), *α*-Phellandrene (1.4%) |
| Cajeput  (*Melaleuca leucadendra*) | Leaves | Eucalyptol (71.8%), α-Pinene (12.0), Limonene (4.4%), α-Terpineol (4.0%), *β*-Pinene (1.3%), Triacetin (1.3%), *γ*-Terpinene (0.7%), *β*-Caryophyllene (0.5%), *α*-Myrcene (0.4%), 3-Carene (0.3%), Terpinen-4-ol (0.3%), *α*-Terpinolene (0.2%), NA (2.6%) |
| Garlic  *(Allium sativum)* | Bulb | Diallyl disulfide (54.6%), Diallyl trisulfide (30.9%), Diallyl sulfide (9.5%), Diallyl tetrasulfide (5.0%) |
| Black pepper  *(Piper nigrum)* | Fruit | Limonene (31.1%), *β*-Caryophyllene (20.9%), β-Pinene (19.9%), 3-Carene (11.6%), *α*-Pinene (9.5%), β-Myrcene (2.5%), *α*-Phellandrene (2.3%), *α*-Copaene (1.6%), α-Caryophyllene (0.6%) |
| Peppermint  *(Mentha × piperita L.)* | Whole plant | Menthone (41.9%), Menthol (32.8%), Menthyl acetate (5.6%), Isomenthol (4.8%), Limonene (4.3%),  *α*-Terpineol (1.9%), *α*-Pinene (1.8%), *β*-Pinene (1.4%), Isopulegol (0.9 %), Piperitone (0.9%), 3-Octanol (0.5%),  *β*-Myrcene (0.3%), Sabinene (0.2%), *β*-Caryophyllene (0.5%), NA (2.3%) |
| Tea tree  *(Melaleuca alternifolia)* | Leaves | Terpinen-4-ol (45.2%), *β*-Pinene (20.9%), *γ*-Terpinene (14.7%), Limonene (7.0%), Cymol (5.6%), *α*-Terpinolene (2.6%), *α*-Terpinene (1.8%), α-Terpineol (1.7%), Thujene (0.7%) |
| Cinnamon  *(Cinnamomum zeylanicum)* | Bark | Cinnamaldehyde (91.9%), β-Caryophyllene (2.8%),  Acetic acid (2.3%), cinnamyl ester (2.3%), Linalool (1.1%)  Cymol (0.8%), Limonene (0.3%), α-Terpineol (0.3%),  Eugenol (0.3%), Benzaldehyde (0.2%) |
| Liquid phytogenic solution (Product A) | NA | Carvacrol (80 mg/l), Cinnamaldehyde (3 mg/l) |

NA=Not applicable.
