# Supplementary material for: Minimum inhibitory concentrations of commercial essential oils against common chicken pathogenic bacteria and their relationship with antibiotic resistance
Source: J Appl Microbiol. 2021 Sep 28;132(2):1025–35. doi: 10.1111/jam.15302 (PMC9293407; doi:10.1111/jam.15302)
Supplement: Supplementary file 2 — Table S2 [file JAM-132-1025-s001.docx]

**Table S2**. Median [and interquartile range, IQR] values of MIC of 8 EOs obtained for 47 bacterial pathogens belonging to 5 species.

|  | ORT | *P. multocida* | *A. endocarditidis* | *G. anatis* | *E. coli* | All pathogens |
| --- | --- | --- | --- | --- | --- | --- |
| Cinnamon | 1.0 [0.5-2.0] | 2.0 [2.0-4.1] | 1.0 [0.5-2.0] | 0.3 [0.3-1.0] | 0.3 [0.3-0.35] | 0.5 [0.3-2.0] |
| Product A | 1.9 [1.0-1.9] | 3.8 [1.9 -3.8] | 7.7 [3.8-7.7] | 3.8 [0.8-7.7] | 3.8 [3.3-3.8] | 3.8 [1.9-3.8] |
| Oregano | 5.7 [1.9-60.8] | 60.8 [60.8-121.5] | 60.8 [26.6-121.5] | 11.4 [6.7-60.8] | 30.4 [15.2-53.2] | 30.4 [7.6-60.8] |
| Garlic | 5.9 [1.6-376.9] | 31.6 [2.0-63.1] | 7.9 [1.8-221.0] | 23.7 [7.9->505.0] | >505.0 [>505.0->505.0 ] | 63.1 [3.9->505.0 ] |
| Tea tree | 41.2 [10.7-137.4] | 219.8 [219.8-219.8] | 219.8 [109.9-219.8] | 164.9 [109.9-219.8] | 439.5 [219.8->439.5] | 219.8 [109.9-219.8] |
| Peppermint | 1.7 [0.9-9.6] | 1.7 [1.7-223.0] | 223.0 [87.1-223.0] | 223.0 [195.1->446.0] | >446.0 [>446.0->446.0] | 223.0 [1.7->446.0] |
| Cajeput | 14.2 [1.6-227.5] | 455.0 [113.8-750.0] | 455.0 [227.5->455.0] | 455.0 [199.1->455.0] | >455.0 [>455.0->455.0] | 455.0 [113.8->455.0] |
| Black pepper | 80.9 [5.9->431.5] | 215.8 [215.8->431.5] | >431.5 [377.6->431.5] | >431.5 [>431.5->431.5] | >431.5 [>431.5->431.5] | >431.5 [215.8->431.5 ] |
| All EOs | 3.5 [1.7-54.9] | 63.1 [2.0-219.8] | 110.7 [7.7-227.5] | 110.7 [7.6->431.5] | 627.5 [4.8-750.0] |  |
